# Supplementary material for: The interactome of intact mitochondria by cross-linking mass spectrometry provides evidence for coexisting respiratory supercomplexes
Source: Mol Cell Proteomics. 2017 Dec 8;17(2):216–32. doi: 10.1074/mcp.RA117.000470 (PMC5795388; doi:10.1074/mcp.RA117.000470)
Supplement: Supplemental Data [file supp_RA117.000470_133922_0_supp_23978_fzffwf.zip › spectra_annotation/mito_DR_spectra_annotation/81-1-5-1-9-1.pdf]

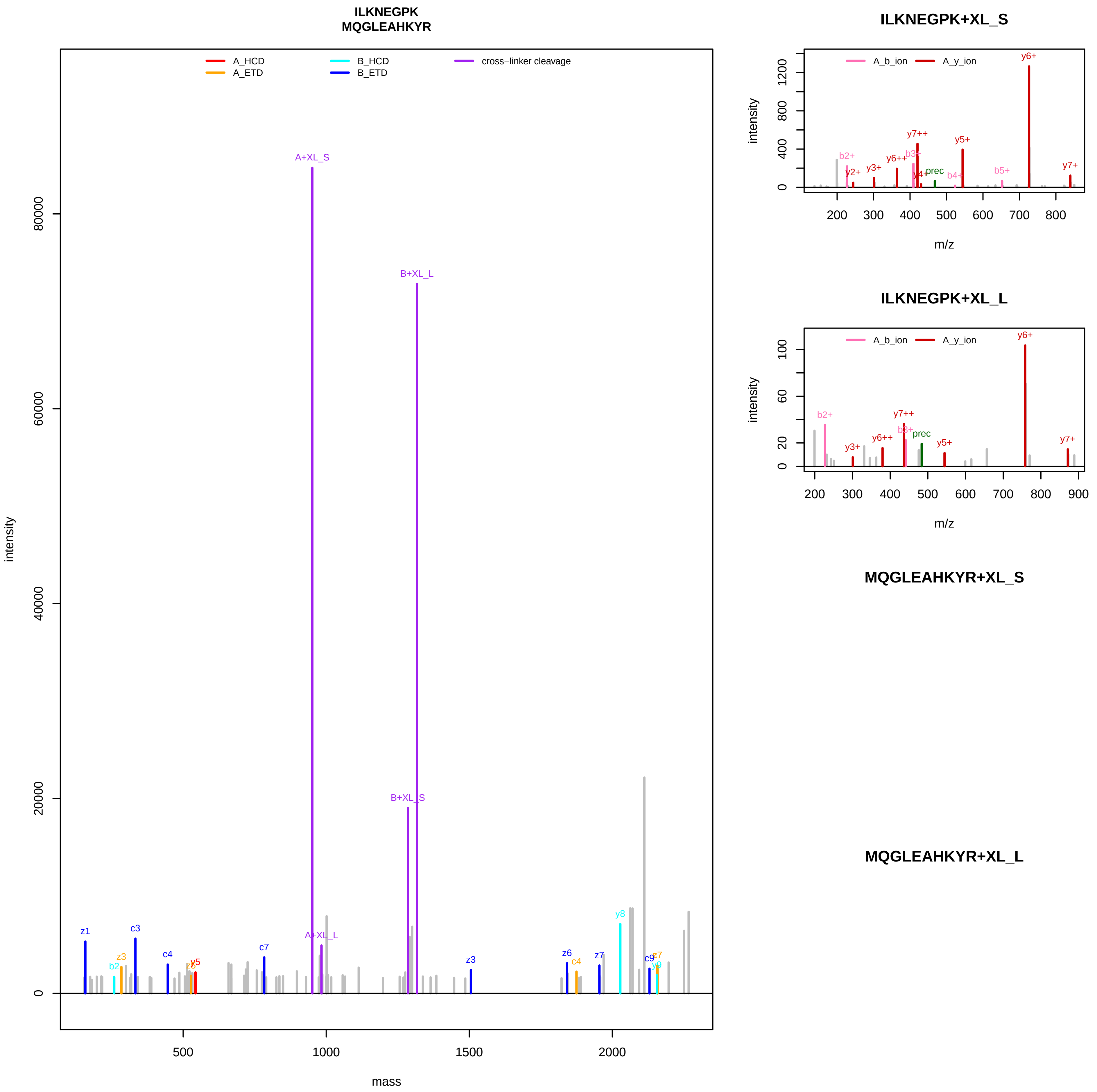

ILKNEGPK
MQGLEAHKYR
A_HCD
A_ETD
B_HCD
B_ETD
cross−linker cleavage
A+XL_S
B+XL_L
B+XL_S
A+XL_L
z1 b2 z3 c3 c4 z3 y5 c7 z3 z6 c4 z7 y8 c9 y9 z7
intensity
mass
0
20000
40000
60000
80000
500
1000
1500
2000
ILKNEGPK+XL_S
A_b_ion
A_y_ion
b2+ y2+ y3+ y6++ y7++ b3+ y4+ prec b4+ y5+ b5+ y6+ y7+
m/z
ILKNEGPK+XL_L
b2+ y3+ y6++ y7++ b3+ prec y5+ y6+ y7+
MQGLEAHKYR+XL_S
MQGLEAHKYR+XL_L
